# Pubertal Expression of α4βδ GABA_A_ Receptors Reduces Seizure-Like Discharges in CA1 Hippocampus

**DOI:** 10.1038/srep31928

**Published:** 2016-08-26

**Authors:** Lie Yang, Hui Shen, Lisa R. Merlin, Sheryl S. Smith

**Affiliations:** 1 Department of Physiology & Pharmacology, SUNY Downstate Medical Center, Brooklyn, NY 11203, USA; 2 Department of Biomedical Engineering, Tianjin Medical University, 22 Qixiangtai Road, Heping District, Tianjin 30070, China; 3 Department of Neurology, SUNY Downstate Medical Center, Brooklyn, NY 11203, USA; 4 Department of Neurology, Kings County Hospital, Brooklyn, NY 11203, USA.

## Abstract

More than half of children with epilepsy outgrow their seizures, yet the underlying mechanism is unknown. GABAergic inhibition increases at puberty in female mice due to expression of extrasynaptic α4βδ GABA_A_ receptors (GABARs). Therefore, we tested the role of these receptors in regulating seizure-like discharges in CA1 hippocampus using a high K^+^ (8.5 mM) seizure model. Spontaneous field potentials were recorded from hippocampus of pre-pubertal (~28–32 PND) and pubertal (~35–44 PND) female wild-type or α4−/− mice. The coastline length, a measure of burst intensity, was assessed. 8.5 mM K^+^ induced seizure-like discharges in over 60% of pre-pubertal slices, but only in 7% of pubertal slices, where the coastline length was reduced by 70% (P = 0.04). However, the pubertal decrease in seizure-like discharges was not seen in the α4−/−, implicating α4βδ GABARs as the cause of the decreased seizure-like activity during puberty. Administration of THIP or DS2, to selectively increase α4βδ current, reduced activity in 8.5 mM K^+^ at puberty, while blockade of α5-GABARs had no effect. GABAergic current was depolarizing but inhibitory in 8.5 mM K^+^, suggesting a mechanism for the effects of α4βδ and α5-GABARs, which exhibit different polarity-dependent desensitization. These data suggest that α4βδ GABARs are anti-convulsant during adolescence.

Epilepsy is a common neurological disorder in childhood. Epilepsy is more than twice as common in children as in adults (about 700 per 100,000 in children under the age of 16 years compared to 330 per 100,000 in adults)^1^. Furthermore, the incidence of status epilepticus in developed countries is between 17 and 23/100,000, with a higher incidence in younger children^1^. These skewed statistics reflect the fact that epilepsy frequently remits in adolescence^2^, an outcome which is more commonly noted for certain epilepsy syndromes^3^. However, the underlying mechanism for this marked decrease in seizure susceptibility during adolescence is not known.

One factor that has not yet been examined is the role of extrasynaptic GABAergic inhibition, which increases at the onset of puberty^4^. GABA_A_ receptors (GABARs) are pentameric membrane proteins of diverse subtype which gate a Cl^−^ conductance and are the most prevalent source of inhibition in the brain^5^. They are localized not only sub-synaptically, but certain subtypes, such as α4βδ, localize at extrasynaptic sites^6^ where they generate a tonic inhibition from ambient GABA due to their high affinity for GABA and relative lack of desensitization^7^ under steady-state conditions. Highest levels of expression of α4βδ GABARs are found in dentate gyrus granule cells^8^, where they generate a tonic inhibition^9^. However, the CA1 hippocampus, which normally exhibits very low levels of expression of this receptor exhibits a dramatic increase in expression during the pubertal period^4^,^10^. Previous studies from this laboratory have shown that expression of α4βδ GABARs increases 4 to 8-fold on the dendrites of CA1 hippocampal pyramidal cells at the onset of puberty in female mice from nearly undetectable levels assessed pre-pubertally^4^,^11^. This increase in tonic inhibition reduces neuronal excitability at puberty^4^. One would expect this increase in GABAergic inhibition during puberty to have an inhibitory effect on CA1 hippocampal network activity such as the production of epileptiform bursts. Therefore, we tested whether pubertal CA1 hippocampus would generate less seizure-like activity than pre-pubertal hippocampus, and we examined the influence of various GABAR subtypes on spontaneous discharge in high K^+^ during puberty to test the hypothesis that α4βδ GABARs selectively reduce seizure-like activity at puberty.

To test neuronal excitability in the hippocampus, we exposed hippocampal slices to high K^+^ to elicit synchronized bursting in the CA1 region. This is a well-established *in vitro* model commonly used to elicit seizure-like activity^12^,^13^,^14^. The magnitude of the resultant epileptiform activity was assessed using the coastline length measurement, a measure of individual burst intensity (the summation of burst amplitude, duration and intra-burst frequency)^12^, across pubertal stages in wild-type and α4−/− mice.

## Results

### Pubertal slices are resistant to expression of seizure-like discharges

**Figure 1:**
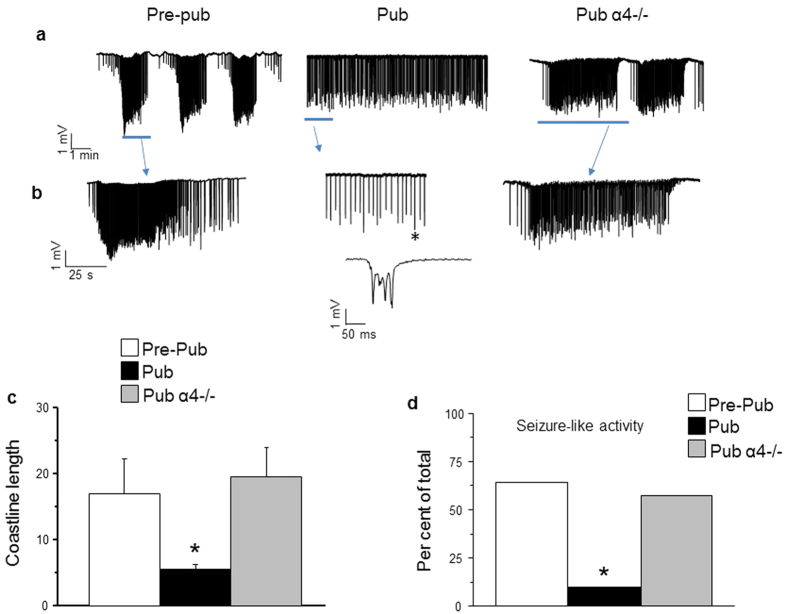
A high K^+^ seizure model triggers less seizure-like activity at puberty: Reversal by α4 knock-out. (**a**) Representative 20 min traces illustrating the predominant response to 8.5 mM K^+^ for each group. *Left*, pre-pubertal WT, *middle*, pubertal WT, *right*, pubertal α4−/−. (**b**) Expansion of the time-base for indicated regions (blue arrow). (**c**) Averaged coastline length, a measure of neuronal activity, reveals greater activity pre-pubertally and in the pubertal α4−/−. *ANOVA, F(2, 32) = 3.50, P = 0.042 vs. other groups (100 s after 8.5 mM K^+^). (**d**) Percent of slices exhibiting seizure-like activity. *P = 0.005 vs. other groups, χ^2^  =  10.6, DF = 2. (n = 7 mice, α4−/−; n = 14 mice, WT Pre-pub; n = 14 mice, WT Pub).

We perfused slices with high K^+^ (8.5 mM) to elicit seizure-like discharges^12^ and compared the response in pubertal vs. pre-pubertal hippocampus. All slices continuously bathed in 8.5 mM K^+^ developed a pattern of spontaneous synchronized bursting, the magnitude of which increased gradually, stabilized by 35–50 min and could be maintained for hours. Two types of discharges were observed: seizure-like (“ictal activity”) or synchronized discharge (“inter-ictal activity”)^15^. Seizure-like activity was observed in over 60% of the pre-pubertal hippocampal slices (9 out of 14 slices) but in only 7% of the pubertal slices (1 out of 14 slices, P = 0.005, Fig. 1) in 8.5 mM K^+^. The remainder of the pre-pubertal slices exhibited discharge patterns similar to the typical pubertal pattern. In addition, the coastline length was reduced by 70% at puberty compared to pre-pubertal values (pre-pub, 16.8 ± 5.2, pub, 5.4 ± 0.78, P = 0.04, Fig. 1), suggesting that increases in synaptic activity produced by the high K^+^ aCSF are reduced at puberty.

### Reduced expression of seizure-like discharges at puberty is not seen in the α4 knock-out

Because α4βδ GABARs are increased at puberty^4^,^10^, we tested whether knock-out of α4 would increase seizure-like activity at this time. To this end, we evaluated the coastline length for pubertal α4−/− hippocampal activity in 8.5 mM K^+^. This parameter was increased more than 100% above pubertal wild-type (WT) values (19.3 ± 4.4, P = 0.04, Fig. 1), and was not significantly different from pre-pubertal values. Seizure-like activity was observed in nearly 60% of the slices (4 out of 7 slices), similar to pre-pubertal assessments, and significantly more likely to occur than in pubertal wild-type slices (P = 0.005, Fig. 1). These data suggest that the increased expression of α4βδ GABARs at puberty exerts an anti-convulsant effect.

### Increasing α4βδ GABAR-gated current with THIP further reduces spontaneous discharge in high K^+^ at puberty

**Figure 2:**
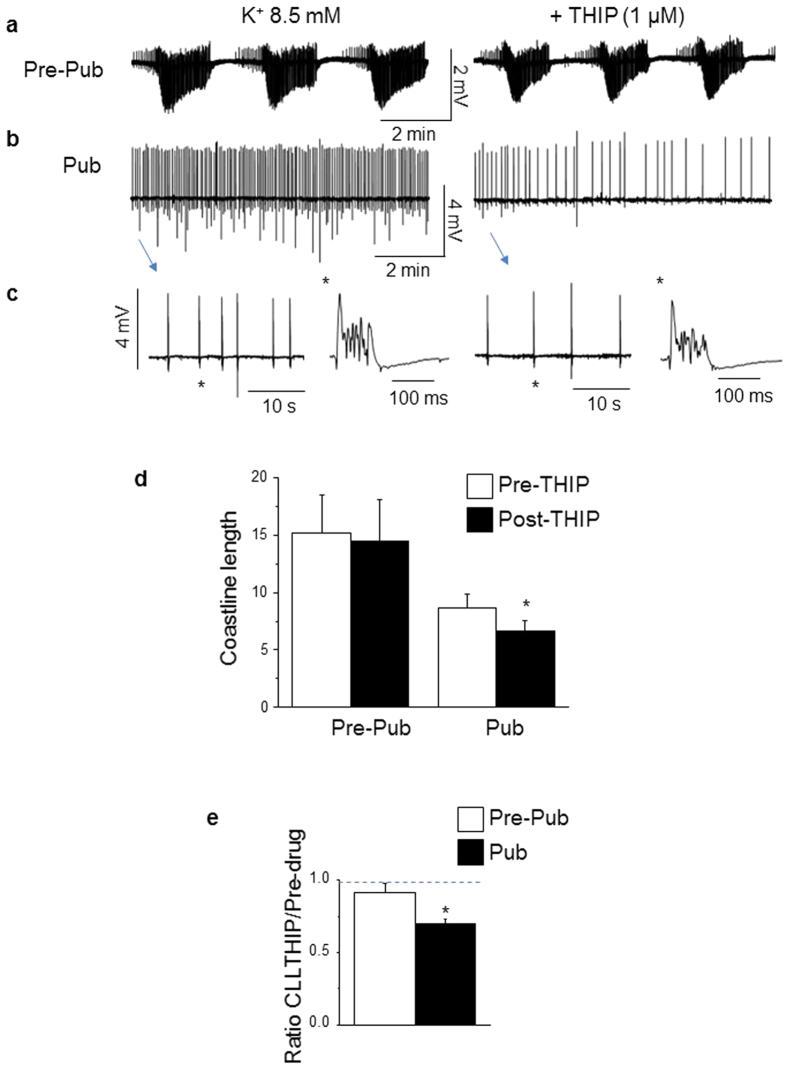
THIP selectively reduces spontaneous discharge in high K^+^ at puberty. (**a,b**) Representative traces of spontaneous synchronized discharges recorded in 8.5 mM K^+^ from pre-pubertal (**a**) and pubertal (**b**) CA1 hippocampus before and after 1 μM THIP, which is selective for δ-GABARs. (**c**) Expanded time-base of traces (Pub) presented in (**b**). (*individual event from indicated trace) (**d**), Averaged values of the coastline length, ^#^slices/group, n = 19 slices from 10 mice, Pre-pub; n = 15 slices from 10 mice, Pub. *(1-tail) paired t-test, t(14) = 7.26, P = 2.07 × 10^−6^ vs. pre-THIP. (**e**) Averaged ratios of the coastline length (CLL) for THIP relative to the pre-drug control. (2-tail) t-test, t(32) = 3.46, *P = 0.0016 vs. pre-pub.

Because α4 knock-out increased seizure-like activity and the coastline length in pubertal hippocampus to pre-pubertal levels, we tested the effects of the GABA agonist THIP at a concentration selective for δ-containing GABARs^7^,^16^. We predicted that THIP would have a greater effect to reduce synaptic activity in pubertal hippocampus compared to pre-pubertal. Synchronized activity was recorded from CA1 hippocampus (pre-pubertal and pubertal) in 8.5 mM K^+^ before and after bath application of 1 μM THIP. THIP produced significant decreases in both the frequency (P = 0.0023) and amplitude (P = 2.5 × 10^−5^) of spontaneous events (Table 1), resulting in a 24% decrease in the coastline length (pre-THIP, 8.7 ± 1.2; post-THIP, 6.6 ± 1; Fig. 2, P = 2.07 × 10^−6^). In contrast, THIP had no significant effect on the coastline length in pre-pubertal hippocampus (pre-THIP, 15.2 ± 3.36; post-THIP, 14.5 ± 3.7; P = 0.24, Fig. 2). These results are consistent with the finding that the α4βδ GABARs exert anti-convulsant effects selectively at puberty, when expression of this receptor subtype is increased.

### Increasing α4βδ GABAR-gated current with DS2 further reduces spontaneous discharge in high K^+^ at puberty

**Figure 3:**
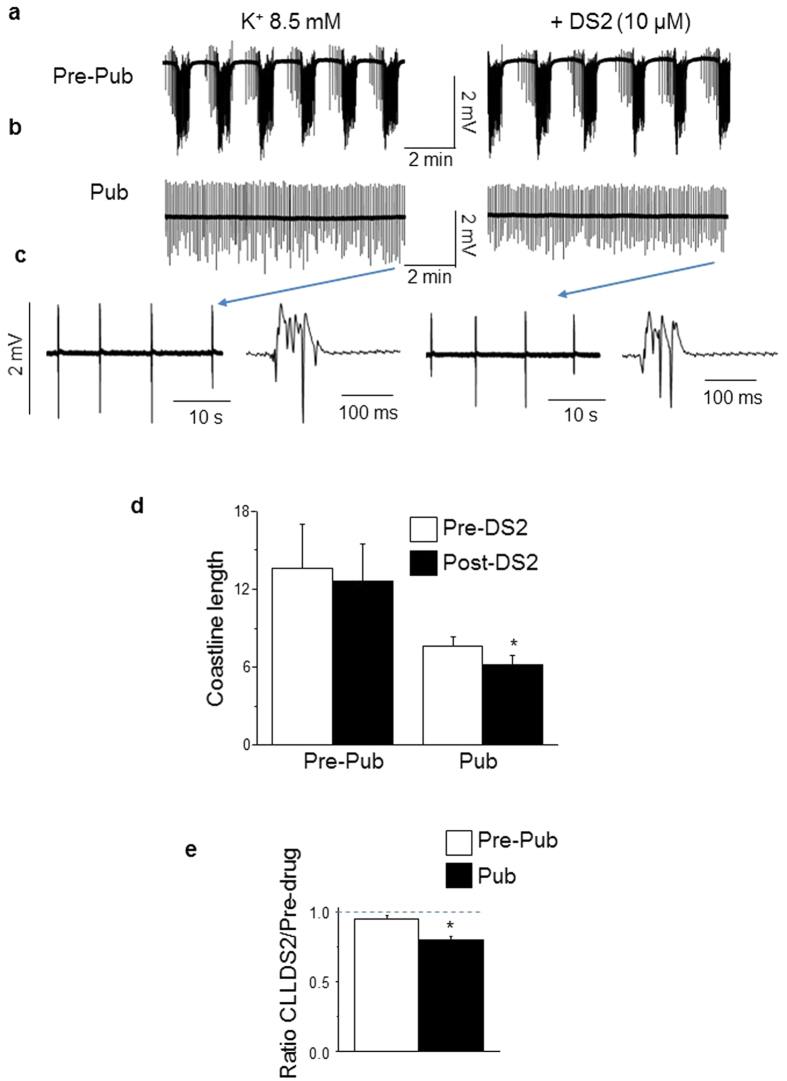
DS2 selectively reduces spontaneous discharge in high K^+^ at puberty. (**a,b**) Representative traces of neuronal activity recorded in 8.5 mM K^+^ from pre-pubertal (**a**) and pubertal (**b**) CA1 hippocampus before and after 10 μM DS2, a GABA modulator which is selective for δ-GABARs. (**c**) Expanded time-base of traces (Pub) presented in (**b**). (*individual event from indicated trace) (**d**), Averaged values of the coastline length, *(1-tail) paired t-test, t(16) = 6.8, P = 2.15 × 10^−6^ vs. pre-DS2. (**e**) Averaged ratios of the coastline length (CLL) for DS2 relative to the pre-drug control. *(2-tail) t-test, t(33) = 3.85, P = 5.1 × 10^−4^ vs. pre-pub. n = 18 slices from 9 mice, Pre-pub; n = 18 slices from 9 mice, Pub.

We compared effects of another δ–selective GABA drug^17^, DS2 (10 μM), on epileptiform activity in high K^+^ aCSF at puberty. We also predicted that DS2 would have a greater effect to reduce synaptic activity in pubertal hippocampus compared to pre-pubertal. In fact, DS2 produced a significant 17% decrease in the coastline length (pre-DS2, 7.62 ± 0.7; post-DS2, 6.3 ± 0.7; Fig. 3, P = 2.2 × 10^−6^) at puberty, due to significant decreases in both the frequency (P = 0.00024) and amplitude (P = 1.29 × 10^−6^) of spontaneous events (Table 1). In contrast, bath application of this drug had no significant effect on the coastline length in pre-pubertal hippocampus (pre-DS2, 13.6 ± 3.4; post-DS2, 12.6 ± 2.9; P = 0.10, Fig. 3). These results are consistent with the finding that the α4βδ GABARs exert anti-convulsant effects selectively at puberty.

### Blockade of α5-GABARs does not increase spontaneous discharge in high K^+^at puberty

**Figure 4:**
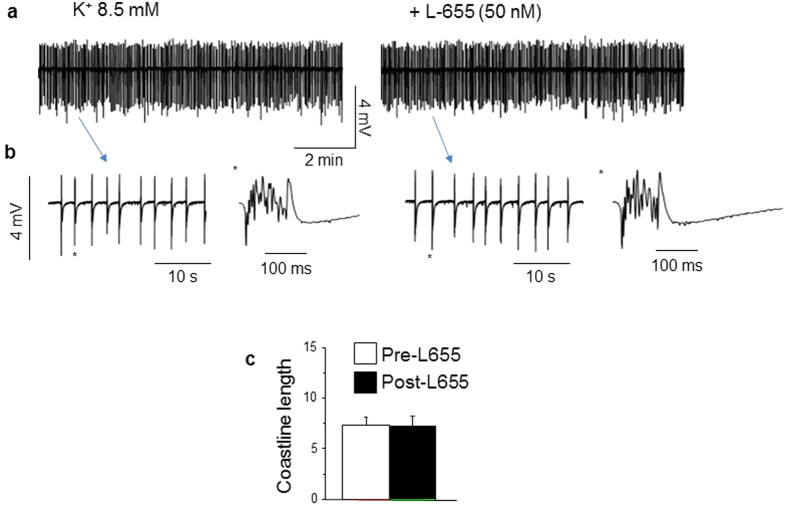
Blockade of α5-GABARs has no effect on spontaneous discharge in high K^+^ at puberty. (**a**) Representative traces of spontaneous synchronized activity recorded in 8.5 mM K^+^ from pubertal CA1 hippocampus before (left) and after (right) 50 nM L-655,708 (L-655) to block α5-GABARs. (**b**) Expanded time-base of the traces presented in (**a**). (*individual event from indicated trace) (**c**), Averaged values of the coastline length. (**d**) Averaged ratios of the coastline length (CLL) after L-655 relative to the pre-drug control. n = 12 slices from 6 mice/group.

α5βγ2 GABARS are the predominant extrasynaptic GABAR subtype in the CA1 hippocampus where they generate a tonic inhibitory current^18^. Therefore, we tested whether blockade of this receptor with the selective inverse agonist, L-655,708^18^, would alter synaptic activity at puberty in 8.5 mM K^+^. To this end, neuronal activity was recorded in pubertal slices before and after bath application of 50 nM L-655,708 (IC_50_ = 20 nM)^16^. L-655,708 produced no significant change in the coastline length (pre-L-655,708, 7.3 ± 0.8; post-L655,708, 7.2 ± 1; P = 0.75, Fig. 4) or in the frequency or amplitude of spontaneous events (Table 1), suggesting that α5βγ2 GABARs do not contribute to the reduced seizure-like activity observed at puberty.

### Blockade of synaptic GABARs only moderately increases spontaneous discharge in high K^+^ at puberty

**Figure 5:**
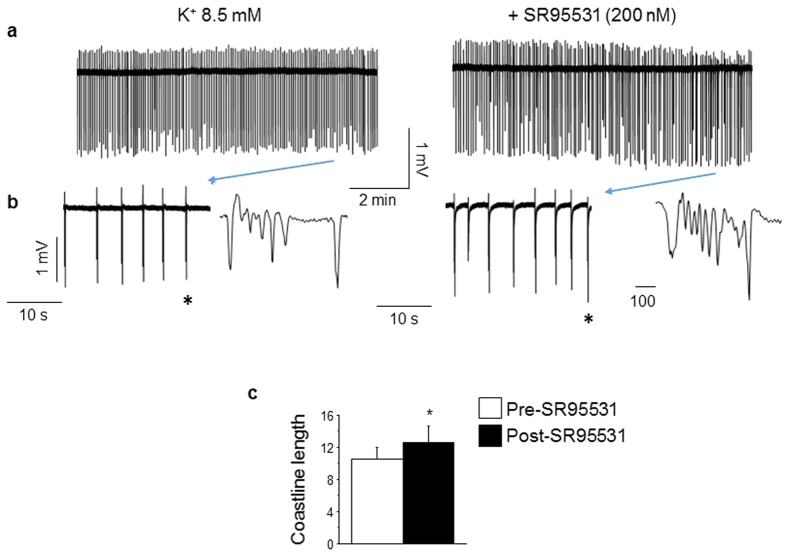
Blockade of synaptic GABARs increases spontaneous discharge in high K^+^ at puberty. (**a**) Representative traces of spontaneous synchronized activity recorded in 8.5 mM K^+^ from pubertal CA1 hippocampus before (left) and after (right) 200 nM SR95531 to block synaptic GABARs. (**b**) Expanded time-base of the traces presented in (**a**). (*individual event from indicated trace) (**c**), Averaged values of the coastline length. (**d**) Averaged ratios of the coastline length (CLL) after SR95531 relative to the pre-drug control. *(1-tail) paired t-test, t(15) = 2.52, P = 0.024 vs. pre-SR95531, n = 16 slices from 8 mice/group.

We tested the role of synaptic GABARs in modulating epileptiform activity of pubertal hippocampus in high K^+^ aCSF. To this end, neuronal activity was recorded in pubertal slices before and after bath application of 200 nM SR95531 (gabazine). At this concentration SR95531 exerts an almost complete block of the synaptic GABAR population, selectively^4^,^9^. Synaptic GABAR blockade produced a significant reduction in burst amplitude (P = 0.04) but not burst frequency (Table 1). Analysis of coastline length revealed only an 18% increase (pre-SR95531, 10.55 ± 1.5; post-SR95531, 12.5 ± 2.1; P = 0.024, Fig. 5), suggesting that synaptic GABARs have a lesser role in suppressing seizure discharges at puberty than α4βδ GABARs.

### Cell-attached recordings of spontaneous activity in high K^+^ aCSF across pubertal state

**Figure 6:**
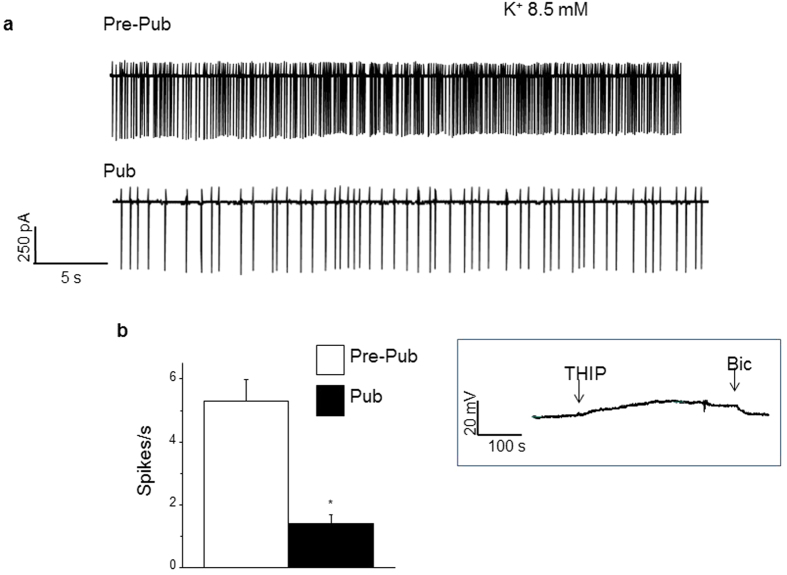
High K^+^ triggers less spontaneous discharge at puberty: Cell-attached recordings. Loose seal, cell-attached recordings from CA1 hippocampal pyramidal cells (taken with the amplifier in voltage clamp mode) reveal a greater rate of spontaneous spiking in 8.5 mM K^+^ before puberty (Pre-pub, upper trace) compared to puberty (Pub, lower trace). (**a**) Representative traces. (**b**) Averaged data. t-test (2-tailed), t(9) = 5.7, *P = 2.8 × 10^−4^; n = 5–6 mice/group. Inset, current through GABA_A_ receptor-channels is depolarizing in 8.5 mM K^+^ aCSF. Use of tight-seal, cell-attached recording of a CA1 hippocampal pyramidal cell (taken with the amplifier in current clamp mode) reveals a depolarization of the membrane potential upon application of 5 μM THIP (first arrow) to the slice, which repolarized to baseline upon application of 20 μM bicuculline (second arrow). This depolarization indicates outward Cl^−^ flux. (Representative of 6 cells (1 cell/mouse)).

In order to determine the activity level of an individual pyramidal cell, we used loose seal cell-attached voltage clamp recordings^4^ to compare spontaneous activity generated in 8.5 mM K^+^ in pre-pubertal versus pubertal hippocampus. This technique is advantageous in that it permits assessment of spontaneous activity without disturbing the intracellular milieu. Spiking was decreased by 75% in 8.5 mM K^+^ at puberty compared to pre-puberty (pre-puberty, 5.3 ± 0.7 spikes/s; puberty, 1.4 ± 0.3 spikes/s; P = 0.00028, Fig. 6), confirming the findings suggested by field recordings that the pubertal hippocampus is less excitable than the pre-pubertal hippocampus in high K^+^ aCSF.

### Determination of the polarity of GABAergic current in high K^+^ aCSF

Because the desensitization of α4βδ and α5βγ2 GABARs are differentially influenced by the polarity of GABA-gated current^19^,^20^, we determined the polarity of the GABAergic current using tight seal, cell-attached current clamp recordings^21^,^22^. Previous studies have suggested that high K^+^ aCSF results in depolarizing GABAergic current, which would selectively accelerate desensitization of α5βγ2 GABARs. For this technique, the use of a >1 GΩ seal and passing 0 current, allows the estimation of the membrane potential change in response to a GABA agonist. Application of the GABA agonist THIP (5 μM) produced an upward deflection (Fig. 6), reflecting outward Cl- flux, that would result in depolarizing GABA current. This deflection returned to baseline upon application of 20 μm bicuculline, verifying the GABAergic nature of the response.

## Discussion

This study demonstrates that seizure-like activity generated by a high K^+^ model is significantly decreased during the pubertal period due to the increased inhibition provided by α4βδ GABARs which emerge during adolescence in CA1 hippocampus^4^,^11^. This decrease in epileptiform activity was not seen in the α4−/− mouse hippocampus confirming the role of the α4βδ GABAR in reducing seizure-like activity. This finding may be relevant for remission of childhood epilepsy in adolescence, which is reported for 50–60% of the cases studied^2^,^23^.

Patients with epilepsy experience recurrent unprovoked seizures, which are the result of a hypersynchronous discharge of a localized network of pyramidal cells^15^,^24^. This seizure discharge would have to last for at least a second or two to result in a clinically-appreciable disturbance, which is why we focused our attention on the presence or absence of long-duration “seizure-like” discharges we observed in our experiments.

In the present study, epileptiform activity was assessed using the coastline length measurement^12^, which incorporates amplitude, duration and frequency of neuronal activity for a given time period. This parameter was markedly reduced in 8.5 mM KCl at puberty for WT but not α4−/− hippocampus compared to the pre-pubertal hippocampus. In addition, however, the pubertal WT hippocampus displayed virtually no seizure-like activity, compared to the pre-pubertal WT and pubertal α4−/− hippocampi, suggesting that it is more resistant to the expression of seizure activity.

Expression of α4βδ GABARs is increased by up to 8-fold at the onset of puberty in the female mouse CA1 hippocampus compared to nearly undetectable levels pre-pubertally^4^,^11^. Expression was established using multiple immunolabeling techniques, including silver-intensified immunogold labeling and electron microscopic quantification, which permits surface localization, while functional expression was confirmed by responses of the tonic current to 100 nM THIP, selective for δ-containing GABARs^7^,^16^, using whole cell voltage clamp recording techniques^4^,^10^. Our previous studies have shown that the presence of this receptor during the pubertal period (PND 35–44) decreases the input resistance of the neuron and reduces spontaneous activity as well as increasing the threshold for generating an action potential^4^. In the present study, the reduced excitability of pubertal CA1 neurons was also observed as reduced single cell spiking compared to pre-pubertal hippocampus in the presence of high K^+^. In all cases, these outcomes were not observed in the α4−/− hippocampus at puberty, confirming the role of the α4βδ GABAR in reducing CA1 pyramidal cell intrinsic excitability at puberty.

GABARs containing the α4 subunit can co-express with γ2 in addition to δ, while δ-containing GABARs can also express with α1^25^. However, at puberty, the majority of receptors which emerge on CA1 hippocampal pyramdiall cells are α4βδ GABARs based on several lines of evidence. First, pubertal pyramidal cells respond robustly to 100 nM THIP, a GABA agonist, which, at this concentration is selective for α4βδ^7^,^16^ (rather than α1βδ)^26^. Secondly, CA1 pyramidal cells of α4−/− mice have significantly reduced surface expression of the GABAR δ subunit and little or no response to 100 nM THIP^27^, suggesting that knock-out of α4 reduces δ expression to produce a functional α4βδ knock-out. This is not surprising as α1 co-expression with δ has only been reported on interneurons^28^, suggesting that this sub-type may not express on pyramidal cells. Thus, the recovery of seizure-like activity in the α4−/− is due to the lack of α4βδ GABARs.

Another factor which may have contributed to the pubertal-associated reduction in seizure activity is the neurosteroid THP (3α-OH-5α-pregnan-20-one), a positive GABAR modulator with greatest effects noted at α4βδ GABARs^29^. Although the day of puberty onset (vaginal opening) is associated with a transient decrease in hippocampal levels of this steroid^4^, circulating levels of THP would be increased after puberty because it is a metabolite of the ovarian steroid progesterone. THP is known to exhibit anti-convulsant effects^30^ as a result of its ability to increase GABAergic inhibition. Although our previous findings suggest that THP can exhibit paradoxical effects to reduce inhibition at α4βδ GABARs, these were observed with hyperpolarizing current gated by these receptors^4^. The depolarizing, inhibitory (shunting) current produced by the high K^+^ seizure model would be increased by THP, and thus would reduce seizure-like activity. We cannot exclude the possibility that other neurotransmitter receptors and ion channels might contribute to the reduced seizure state at puberty, but α4βδ GABARs play a major role because seizure activity was markedly increased in the α4−/− hippocampus.

Other studies have reported increased expression of α4 following pilocarpine injection in a rodent model of temporal lobe epilepsy (TLE)^31^. Increased expression of α4-containing GABARs and reduced expression of α1-GABARs in this model was associated with increased seizure frequency, which could be reduced by increasing expression of α1-GABARs^32^. However, in contrast to the present study, this model of TLE also results in reduced δ expression and increased γ2 expression^33^, suggesting that it increases α4βγ2 GABARs rather than α4βδ GABARs. α4βγ2 GABARs have a faster deactivation^34^, dependent upon the β isoform, and greater desensitization compared to α1βγ2^34^, which would yield less inhibition. For this reason, pubertal reductions in seizure activity may not occur in the dentate gyrus in TLE.

The high K^+^ seizure model has been used previously to demonstrate seizure-like activity^12^,^13^,^14^, which originates from CA3 hippocampal pyramidal cells triggering seizure bursts in CA1 pyramidal cells via the Schaffer collaterals. Elevated K^+^ levels occur in seizure states^36^,^37^, as well as following increases in neuronal activity^38^. Increases in extracellular K^+^ have also been documented to depolarize the membrane and reduce input resistance^39^, leading to increased hyper-excitability.

The high K^+^ model is particularly convenient to use to examine developmental changes because it is induced acutely in the slice preparation. Other models would not be feasible for comparing pre-pubertal and pubertal seizure susceptibility because they require a longer time-course to establish the model and thus would overlap both developmental periods.

GABAergic current recorded in the high K^+^ seizure model at puberty in the present study was a depolarizing, shunting inhibition because depolarization was not sufficient to reach the threshold for triggering an action potential. GABA’s inhibitory effects were observed as a direct reduction in epileptiform activity after bath application of the GABA agonist THIP. However, the current generated by THIP was depolarizing in high K^+^ aCSF. This outcome was observed as a positive deflection of the voltage recorded using tight-seal cell-attached recordings^21^. This is in contrast to the typical hyperpolarizing direction of current normally observed in pubertal hippocampus^4^. The change in polarity observed after exposure to a high K^+^ solution is likely due to reversal of the direction of the KCC2 K^+^-Cl^−^ co-transporter which depends upon a K^+^ gradient^40^,^41^. High internal K^+^ allows for Cl^−^ extrusion, thus maintaining low [Cl^−^]_i_ which produces hyperpolarizing current in response to GABA. However, this function is reversed in the presence of high external K^+^. A number of studies have demonstrated that high K^+^ reduces the E_Cl−_ (less negative) and reverses the direction of the transporter, producing depolarizing GABA responses, which are prevented with furosemide, a KCC2 inhibitor^42^,^43^,^44^.

The pubertal reduction in seizure-like activity was largely mediated by α4βδ GABARs selectively. This was confirmed not only by the increase in seizure-like activity after α4 knock-out, but also by the decrease in spontaneous discharge in high K^+^ produced by δ-selective ligands, THIP and DS2, which did not have significant effects pre-pubertally. Although the effects of these drugs were significant at puberty, they were relatively modest compared to the effect of α4 knock-out. This is likely due to the fact that α4βδ GABARs were close to maximal activation by endogenous GABA, nearly creating a ceiling effect for further anti-convulsant activity by THIP and DS2.

In contrast, blockade of α5β3γ2 GABARs, which account for the majority of extrasynaptic GABARs in CA1 hippocampus^8^,^18^, had no significant effect on spontaneous discharge in high K^+^. This is consistent with other reports demonstrating that L-655,708 is not pro-convulsant at a dose which enhances cognition^45^. The reason for this difference in effect of the two extrasyaptic GABAR subtypes may be due to the fact that these two receptor sub-types exhibit distinct polarity-dependent increases in their rate of desensitization. When GABA-gated current is depolarizing, as in the high K^+^ seizure model, α5β3γ2 GABARs have a faster rate of desensitization^20^, which reduces the steady-state current. The reverse is true for α4βδ GABARs, which desensitize faster and to a greater extent when GABA-gated current is hyperpolarizing^19^, but yield greater steady-state current when GABA is depolarizing. Thus, in this seizure model, α4βδ GABARs would generate more inhibitory current and provide a more effective anti-convulsant effect.

The polarity-dependent properties of α4βδ GABARs may also be important for certain genetic epilepsies which are due to mutations in α4βδ GABARs that reduce their anti-convulsant effect^46^. δ-containing GABARs decrease expression in the dentate gyrus in a mouse model of temporal lobe epilepsy induced by pilocarpine, which increases seizure activity^33^, reflecting the importance of tonic inhibition in reducing seizure activity. However, there are conditions, such as brain trauma, in which GABAergic current becomes depolarizing and excitatory^47^. This outcome would be resistant to the anti-convulsant effects conferred by pubertal expression of α4βδ GABARs.

In contrast to the pronounced effect of α4 knock-out, blockade of the synaptic GABARs had a relatively minor effect on seizure activity (18% versus >100% produced by α4 knock-out) suggesting that tonic inhibition generated by α4βδ GABARs is the primary anti-convulsant mechanism at puberty. This is consistent with reports suggesting that the inhibition produced by the tonic current is greater than that produced by phasic currents^48^.

Remission for childhood epilepsy is currently reported for over 60% of children studied^2^,^23^, an effect which is not gender-specific, suggesting that this is a robust outcome. It is most common in children with an onset of first seizure <10 years of age and a mean age of remission around the time of puberty onset^2^, consistent with the results from the present study. There is a greater probability of remission for uncomplicated, idiopathic seizures which have a frequency of less than 1 seizure/week, unaccompanied by intellectual disability or EEG changes^23^, which are less likely to involve neurodegeneration which may indicate a permanent change in network properties. To date there is no physiological explanation for this finding. Although the most common epilepsies to remit are the primary generalized epilepsies, which do not originate in the temporal lobe, this may be due to the decrease in δ expression in dentate gyrus reported in TLE^33^. Increased δ expression has also been observed in the adolescent cortex^49^ in addition to CA1 hippocampus^10^ suggesting that the anti-convulsant actions of α4βδ GABARs at puberty are site-specific. The present findings suggest a possible mechanism for remission of certain childhood epilepsies and may also suggest possible therapeutic interventions for those epilepsies which do not undergo remission in adolescence.

## Methods

### Animals

Pre-pubertal (~PND 26–32) or pubertal (~PND 35–44) female C57BL6 mice (wild-type (WT) or α4−/−) were used. α4−/− mice were bred from α4+/− (generously supplied by G. Homanics, U. Pittsburgh). Initially, +/+ mice were used, but supplemented with wild-type mice from Jackson labs because they produced similar outcomes to the +/+ mice bred in house. Female mice were used because α4βδ GABAR expression and physiological effects have been well characterized across pubertal development^4^,^10^. Animals were housed in a reverse light:dark cycle vivarium and tested 1 h before the onset of the dark cycle. Puberty onset was verified by vaginal opening^4^. The estrous cycle does not alter GABAergic inhibition during the pubertal period, as we have previously shown^4^. All procedures were approved by and carried out in accordance with the Institutional Animal Care and Use Committee at SUNY Downstate.

### Hippocampal slice preparation

Animals were rapidly decapitated, and the brains removed and cooled using an ice cold solution of artificial cerebrospinal fluid (aCSF) containing (in mM): NaCl 124, KCl 5, CaCl_2_ 2, KH_2_PO_4_ 1.25, MgSO_4_ 2, NaHCO_3_ 26, and glucose 10, saturated with 95% O_2_, 5% CO_2_ and buffered to a pH of 7.4. The hippocampus was removed, and 400–450 μm transverse sections were prepared using a Leica oscillating microtome (cell attached recordings) or a McIlwain tissue chopper (extracellular recordings); slices were then incubated for 1 h in oxygenated aCSF (95% O_2_ and 5% CO_2_). Data were collected from 1–2 slices/animal.

### Electrophysiology-extracellular field recordings

The slices were initially perfused in an interface configuration for >1 h as the bath temperature was raised to 30 ± 1 °C. During the recording period, the submerged configuration was used to permit optimal solution exchange. The control aCSF contained (in mM): NaCl 124, KCl 5, NaH_2_PO_4_ 1.25, NaHCO_3_ 26, CaCl_2_ 2, MgCl_2_ 1.6, and glucose 10 (pH 7.4).

Extracellular recordings were made from the CA1 pyramidal cell body layer using borosilicate glass pipets filled with aCSF (resistance 2–3 MΩ). Spontaneous activity was recorded with AxoScope (MiniDigi 1B, pClamp 10.1, 1 kHz sampling rate) after continuous bath perfusion of 8.5 mM KCl for at least 1 h to elicit a stable pattern of epileptiform activity^12^. For some experiments, GABAR subunit selective drugs were bath applied following stable baseline recordings in the presence of 8.5 mM KCl and spontaneous activity recorded.

### Electrophysiology-cell attached

Pyramidal cells in CA1 hippocampal slice were visualized using a Leica differential interference contrast (DIC)-infrared upright microscope. Recordings were carried out at 22–24 °C using an Axopatch 200B amplifier, at a 10-kHz sampling frequency (2 kHz 4-pole Bessel filter) and pClamp 9.2 software (Molecular Devices, Sunnyvale, CA). Patch pipets were fabricated from borosilicate glass using a Flaming-Brown puller (Sutter Instruments, Novato, CA, USA) to yield open tip resistances of 2–4 MΩ. The bath contained (in mM): NaCl 124, KCl 5, CaCl_2_ 2, KH_2_PO_4_ 1.25, MgSO_4_ 2, NaHCO_3_ 26, and glucose 10, saturated with 95% O_2_/5% CO_2_ and buffered to a pH of 7.4.

### Cell-attached recordings of pyramidal cell spiking

In some cases, loose seal cell-attached recordings were made with the amplifier in voltage clamp mode to assess cell spiking before and after bath application of 8.5 mM KCl, used as a seizure model. The pipet solution contained 150 mM NaCl. The command potential was set to the potential at which the holding current was 0 pA to avoid direct cell stimulation by the electrode^20^. This technique is advantageous in permitting the evaluation of neuronal excitability without disturbing the internal Cl^−^ milieu^4^.

### Cell-attached recording of polarity of GABA_A_ receptor-channel-mediated potential

We used tight-seal cell-attached recording techniques with the amplifier in current clamp mode^21^,^22^ to assess whether the GABA agonist THIP yielded a hyperpolarizing or depolarizing potential in hippocampal CA1 pyramidal cells in slices from pre-pubertal vs. pubertal mice. With a >1 GΩ seal and passing 0 current, R_seal_ ≫ R_patch+cell_ allows determination of the direction of potential change in response to GABA^20^ or in response to an exogenously-applied GABA agonist, as we have previously demonstrated^4^. Therefore, for this study, tight-seal (>1 GΩ) cell-attached recording of membrane potentials were made from the soma of CA1 hippocampal pyramidal cells. The pipet solution contained 150 mM NaCl. The response of the membrane potential to bath application of 5 μM THIP was recorded (with no current injected). For this study, the bath contained tetrodotoxin (TTX, 0.5 μM) to isolate the post-synaptic component and 2 mM kynurenic acid to pharmacologically isolate the GABAergic current.

### Drugs

For some experiments, GABAR subunit selective agents were bath applied following stable baseline recordings in the presence of 8.5 mM KCl in order to determine potential mechanisms for pubertal decreases in seizure-like activity. These agents include: DS2 (4-Chloro-*N*-[2-(2-thienyl)imidazo[1,2-*a*]pyridin-3-yl]benzamide, 10 μM), a positive GABAR modulator selective for α4βδ GABARs^50^, THIP (gaboxadol or 4,5,6,7-tetrahydroisoxazolopyridin-3-ol, 1 μM), a GABAR agonist selective for α4βδ GABARs at 1 μM^7^,^16^, L-655,708 (11,12,13,13a-Tetrahydro-7-methoxy-9-oxo-9*H*-imidazo[1,5-*a*]pyrrolo[2,1-*c*][1,4]benzodiazepine-1-carboxylic acid, ethyl ester, 50 nM), an inverse agonist at α5βγ2 GABARs^18^, and SR-95531 (gabazine or 6-Imino-3-(4-methoxyphenyl)-1(6*H*)-pyridazinebutanoic acid hydrobromide), a GABA antagonist which selectively blocks the phasic current up tp 90% at 200 nM^4^,^9^. All GABAR agents were obtained from Tocris Bioscience. All other chemicals were from Sigma Chemical Co.

### Data analysis

Spontaneous field potentials were detected using a threshold delimited event detection program in pClamp 10.1. In extracellular field recordings of spontaneous discharge, an event was defined as a deflection >0.025 mV from baseline which recovered to baseline. Seizure-like (“ictal”) discharges were defined as synchronous, large amplitude (>2 mV), high frequency (>2/s) events of >2 s duration^15^. Slices which did not exhibit seizure-like activity in 8.5 mM K+ displayed synchronized discharge with reduced frequency, amplitude and duration (“inter-ictal” activity) compared to the seizure-like activity described above.

Epileptiform activities recorded from field recordings are irregular and difficult to quantify by conventional methods; therefore, the coastline length (CLL)^12^ was calculated because it more accurately reflects the strength of such activity. CLL is the summary of the point-to-point distance of a given segment (e.g, 10 min), which defines the overall intensity of spontaneous, synchronized discharge incorporating amplitude and frequency (minus the noise). The MiniAnalysis program (Synaptosoft, Inc., Decatur, GA) was used to calculate the CLL to compare neuronal activity across groups. In cases where drugs were applied directly to the slice, frequency and peak amplitude measures of field potentials were also calculated before and after drug application.

Clampfit was used to analyze the frequency and amplitude of the epileptiform activity as well as to determine the direction of GABAergic current and calculate spike frequency produced by high K^+^ in cell-attached recordings.

### Statistics

Origin (OriginLab, Northampton, MA) was used for all statistical comparisons. Statistical differences among 3 groups were determined using an analysis of variance (ANOVA), followed by post-hoc Tukey’s tests; differences between 2 groups were determined using the Student’s t test or the paired t-test when comparing discharge before and after drug application in the same recording. Differences in the percentage of slices with seizure-like activity were determined using a Chi-square analysis (3 × 2contingency table). Data were shown to fit a normal distribution using the Kolmogorov-Smirnov test for normality. A P < 0.05 was considered as statistically significant. All data are described as the mean ± S.E.M. A description of the statistics used for each experiment is included in the figure legends.

## Additional Information

**How to cite this article**: Yang, L. *et al*. Pubertal Expression of α4βδ GABA_A_ Receptors Reduces Seizure-Like Discharges in CA1 Hippocampus. *Sci. Rep*. **6**, 31928; doi: 10.1038/srep31928 (2016).

## Figures and Tables

**Table 1:** Effects of GABAergic agonists, antagonists and modulators on the frequency and amplitude of spontaneous activity in a high K^+^ seizure model at puberty.

Drug group	n	Frequency	(Hz)	t	P	Amplitude	(mV)	t	P
		Pre-drug	Post-drug			Pre-drug	Post-drug		
THIP	17	0.33 ± 0.04	0.28 ± 0.03	3.3	0.002*	1.64 ± 0.17	1.39 ± 0.17	5.5	<0001*
DS2	24	0.34 ± 0.03	0.32 ± 0.03	4.0	0.0003*	1.27 ± 0.16	1.13 ± 0.16	6.19	<0001*
L655	14	0.43 ± 0.45	0.41 ± 0.43	1.6	0.93	1.53 ± 0.17	1.48 ± 0.19	0.69	0.75
SR95531	16	0.40 ± 0.05	0.39 ± 0.04	0.51	0.69	1.61 ± 0.30	1.97 ± 0.38	2.23	0.04*

Mean ± S.E.M., frequency and amplitude of spontaneous activity recorded before and after bath perfusion of the indicated drugs. δ-selective drugs significantly decreased both the frequency and amplitude of spontaneous spiking, while a selective inverse agonist at α5 GABARs, L-655,708 (L655) had no effect. Selectively blocking synaptic inhibition with 200 nM SR9551 had no effect of frequency but significantly increased the amplitude of spontaneous events.

^*^Denotes statistical significance (1-tail paired t-test).
